# Patient perception on risk of recurrence and decision-making in the management of HER2-positive early breast cancer: Insights from the ASKHER2 European survey

**DOI:** 10.1016/j.breast.2025.104456

**Published:** 2025-03-21

**Authors:** Matteo Lambertini, Christian Jackisch, Olivier Trédan, Maria Vidal, Mário Fontes-Sousa, Antonios Valachis, Rosanna D'Antona, Marcelo Ruz, Eugenia Krone, Miriam Brice, Erwan Berjonneau, Soraia Matos, Olivia Dialla, Laure Guéroult-Accolas

**Affiliations:** a Department of Internal Medicine and Medical Specialties (DiMI), School of Medicine, University of Genova, Genoa, Italy; b Department of Medical Oncology, U.O.C. Clinica di Oncologia Medica, IRCCS Ospedale Policlinico San Martino, Genoa, 16132, Italy; c Kliniken Essen Mitte gGmbH – KEM- Essen, Germany; d Centre Léon Bérard, France; e Cancer Research Center of Lyon (UMR Inserm 1052 – CNRS 5286), France; f IOB Institute of Oncology Barcelona, Spain; g CUF Tejo, Lisboa, Portugal; h Hospital S. Francisco Xavier, ULSLO, Lisboa, Portugal; i Department of Oncology, Faculty of Medicine and Health, Örebro University, Örebro, Sweden; j Europa Donna Italia, Italy; k AMOH, Spain; l Mamazone, Germany; m Careca Power, Portugal; n Cerner Enviza / Oracle, France; o Pierre Fabre Médicament, France; p Patient en Réseau, France

**Keywords:** HER2+, Breast cancer, Risk of recurrence, Patient perception, Communication, Intervention, Decision making

## Abstract

**Background:**

Perceived risk and fear of recurrence in patients with breast cancer (BC) is a matter of concern and may affect their health behaviours and their ability to participate in decision making during their treatment. This survey aimed to examine perceptions and concerns of patients with HER2+ BC.

**Materials and methods:**

A multi-country, non-interventional, direct-to-patient online survey was conducted between July 22, 2022 and March 1, 2023 in six European countries using a multi-modal recruitment approach.

**Results:**

Out of 622 included patients, 96.8 % desired involvement in treatment decisions, and 58.5 % felt they had significant influence in the decision-making process. A total of 20.9 % of patients were unaware of their personal risk of recurrence, and 19.5 % reported not discussing this risk with their healthcare providers. The fear of disease recurrence, death, and treatment failure were identified as the most important concerns. Moreover, 30.4 % perceived they had clear communication with healthcare providers on risk of recurrence. A total of 64.5 % were willing to take extra treatments, 60.2 % to undergo more surgery to reduce recurrence risk and 68.5 % were willing to accept further treatments even if recurrence risk decreased by less than 50 %.

**Conclusion:**

Results of this multinational direct-to-patient study examining the perceptions and concerns of women with HER2+ breast cancer underscore the need for physicians to proactively involve patients in their decision-making process, enabling them to participate in a patient-centred approach during treatment decisions.

## Highlights

• Involvement of patients in the decision making and treatment choices is crucial.
• Physicians should individualize messages depending on each patient's preferences.
• Patients are willing to compromise part of their quality-of-life for a better outcome.
• Psychological insecurities were identified as the most important concerns.
• Effective tools are needed to improve physician-patient communication on risk of recurrence.

## 1 Introduction

Breast cancer (BC) is the most common malignancy among women worldwide, with an estimated incidence of nearly 2.3 million in 2020 [1]. Human epidermal growth factor receptor 2 (HER2) is overexpressed or amplified (HER2+) in about 15 % of invasive BCs and is a well-known negative prognostic factor [2,3]. Over the past years, the rapid progress in targeted therapy for BC and the use of anti-HER2 agents has contributed to an overall 10-year survival rate of more than 80 % in developed countries [4].

Despite HER2-targeted therapies having enhanced outcomes for HER2+ BC, challenges persist [[4], [5], [6]]. Uncertainty about properly estimating the risk of recurrence in individual patients complicates provider-patient communication and may leave women unsure about their individual risk of recurrence and treatment choices [7].

To improve their decision-making process, many women diagnosed with BC expect precise information about their individual risk of recurrence and the available interventions to reduce it, including the benefits and harms of each choice [8]. However, there is a wide variability in the communication of risk estimates among treating physicians as well as uncertainty about prognosis and risk/benefit information. The way physicians communicate risk and uncertainty may influence patients’ perceptions and impact their choices, decision satisfaction and treatment adherence. Specifically, a proper perception about risk of recurrence may motivate or not behaviour to decrease it, thus influencing treatment decision-making which may ultimately affect clinical outcomes [9]. A perceived high risk of recurrence or uncertainty in this regard may lead to increased levels of fear [10]. On the other hand, some degree of fear of cancer recurrence may encourage positive health behaviours such as engagement in treatment planning and adherence, discussion about the risk of recurrence with the medical team, and increased knowledge of the disease [11].

Existing research in this area has often examined the alignment between patients' perceived and actual risk of BC recurrence [12,13]. These studies showed that many patients with BC misperceive their risk of recurrence, either underestimating or overestimating it, and that their perceived risk, fear of recurrence, and uncertainty about prognosis seem to impact their behaviours and decision-making process. While previous studies have explored general BC risk perceptions, there is limited literature on this topic, particularly when considering specifically HER2+ BC. These patients may have different concerns due to the availability of novel agents and new treatment options. To address this gap in knowledge, we conducted a multi-national, direct-to-patient survey involving patients with HER2+ BC aiming to determine patients' perceptions and fears of BC recurrence risk, to describe any additional interventions (including lifestyle choices and supportive interventions) that women with HER2+ BC were willing to undertake to reduce the risk of recurrence, and to assess patients’ willingness to be involved in the decision-making process.

## 2 Material and methods

### 2.1 Study design

A cross-sectional multi-national non-interventional direct-to-patient survey was carried out between July 22, 2022 and March 1, 2023 in six European countries (France, Germany, Italy, Portugal, Spain, and Sweden). A multi-modal recruitment approach, including local patient advocacy groups (PAGs) and patient panels, was used. Prior to survey launch, qualitative cognitive interviews were conducted to pre-test the quality of the questionnaire to ensure that appropriate terminology was used and that patients could understand the questions. The study was approved by the Cerner Enviza/Oracle Life Science Institutional Review Board (IRB).

### 2.2 Population

Patients eligible for inclusion in the survey were women aged 18 years or older, with a self-reported diagnosis of localized/locally advanced or metastatic HER2+ BC (but not those with stage IV de novo disease) that received at least one anti-HER2 therapy. Participants had to have access to an internet-connected mobile device (e.g., smartphone, tablet) or a computer and should have been capable of completing the questionnaire in their local language. All patients provided their consent to participate in the survey.

### 2.3 Recruitment procedure and data collection

Soft quotas were applied to achieve representativeness of the target sample size for each country. Prior to their participation in the survey, potential patients were contacted via an e-mail invitation with a link to an online platform which contained an information letter describing the survey objectives and procedures. Upon consent, patients completed preliminary questions (screener) to determine whether they met the inclusion criteria. The following variables were collected in the screening phase to assess patient eligibility for the survey: sociodemographic characteristics; cancer history; experience with BC. Only patients who fulfilled the inclusion criteria could have access to the questionnaire. The questionnaire was developed in English and translated into the local language of each country by a certified agency. After pre-testing with two respondents per country (except for Sweden where there was one respondent), the questionnaire was updated according to the patients' feedback. The study involved no intervention, invasive research, human testing, or access to subjects’ clinical records for data collection.

Variables concerning worries about the disease and the treatment plan, interaction with healthcare professionals, risk perceptions of recurrence and decision making were collected in the patient questionnaire.

### 2.4 Sample size and statistical analysis

The minimum sample size needed for the survey was estimated using in-house data about the feasibility of recruiting respondents in each country. Accordingly, approximately 600 women (100 from France, 100 from Germany, 100 from Spain, 100 from Italy, 100 from Portugal, and 100 from Sweden) were expected to be recruited for the survey. As the analysis was intended to be descriptive in nature, the estimated sample size was assessed to have a maximum 95 % confidence interval (for a proportion of 50 %) of up to ±4.0 % at a global level and ±9.8 % at a country level. The sample size distribution across countries is presented in Supplementary Table 1 with a total of 622 patients.

Statistical analyses were performed with R software, with RStudio version 1.3.959. Data were summarized using descriptive statistics. Variables were summarized by frequencies and percentages. Comparative analyses between subgroups were performed using Pearson's Chi-squared test or Fisher's exact test. Some analyses were re-run by including only patients with early BC while others compared early vs. metastatic disease, different countries, year of diagnosis, personal risk perception and willingness to be involved in decision making. All p-values were two-tailed and a value of 0.05 or lower was considered statistically significant.

## 3 Results

### 3.1 Sociodemographic and disease-related characteristics

**Fig. 1:**
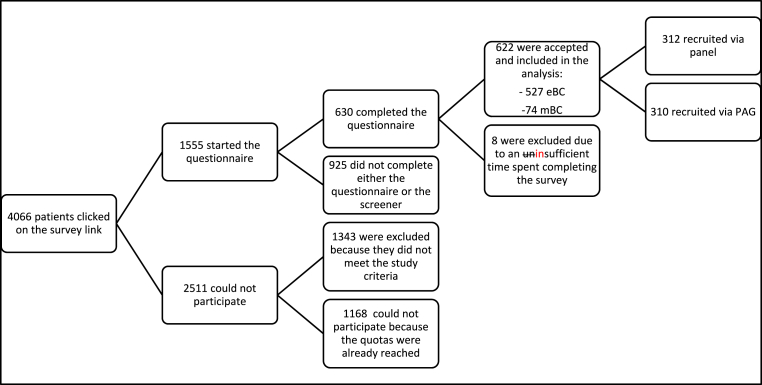
Flowchart of study patients.

In total, 4066 patients entered the survey. Among them, 1168 were excluded due to quotas being full, 925 because they did not fully complete the screener and/or questionnaire, 1343 did not meet the inclusion criteria and eight patients completed the survey in an unrealistically short time interval. The present analysis included 622 patients that met the inclusion criteria and completed the full questionnaire (Fig. 1).

Out of 622 patients, the majority (69.9 %) were between 40 and 65 years of age and 63.5 % were employed. All patients reported having eBC at diagnosis and 84.7 % still had a local BC at the time of the survey, 62.2 % were diagnosed with HER2+ eBC two or more years earlier, 11.9 % self-reported their current BC stage to be metastatic and 67.7 % were undergoing BC treatment at the time of the survey (Table 1).

**Table 1:** Sociodemographic and tumor characteristics of included patients (N = 622).

	Overall (N=622)	France (N=108)	Germany (N=99)	Italy (N=107)	Portugal (N=112)	Spain (N=113)	Sweden (N=83)
**Demographic characteristics**							

Age							
18–39	133(21.4)	11(10.2)	11(11.1)	19(17.8)	35(31.2)	53(46.9)	4 (4.8)
40–65	435(69.9)	78(72.2)	83(83.8)	82(76.6)	75(67.0)	59(52.2)	58(69.9)
>65	54(8.7)	19(17.6)	5(5.1)	6(5.6)	2(1.8)	1(0.9)	21(25.3)
**Current employment**							
Student	7(1.1)	0(0)	0(0)	0(0)	0(0)	5(4.4)	2(2.4)
Employed^a^	395(63.5)	50(46.3)	62(62.7)	77(72)	75(67.0)	83(73.4)	48(57.8)
On leave^b^	64(10.3)	21(19.4)	11(11.1)	3(2.8)	12(10.7)	12(10.6)	5(6.0)
Unemployed	42(6.7)	8(7.4)	3(3.0)	7(6.5)	15(13.4)	8(7.1)	1(1.2)
Retired	89(14.3)	25(23.1)	17(17.2)	12(11.2)	8(7.1)	3(2.7)	24(28.9)
Other^c^	25(4.0)	4(3.7)	6(6.1)	8(7.5)	2(1.8)	2(1.8)	3(3.6)
**Clinical characteristics**							

**Self-reported HER2+ BC**							
Yes	564(90.7)	93(86.1)	97(98.0)	87(81.3)	109(97.3)	100(88.5)	78(94.0)
No	27(4.3)	4(3.7)	0(0)	16(15.0)	1(0.9)	5(4.4)	1(1.2)
Do not know	31(4.9)	11(10.2)	2(2.0)	4(3.7)	2(1.8)	8(7.1)	4(4.8)
**Self-reported current metastatic stage**							
Yes	74(11.9)	11(10.2)	6(6.1)	9(8.4)	27(24.1)	12(10.6)	9(10.8)
No	527(84.7)	96(88.9)	86(86.9)	97(90.7)	81(72.3)	100(88.5)	67(80.7)
Do not know	21(3.4)	1(0.9)	7(7.1)	1(0.9)	4(3.6)	1(0.9)	7(8.4)
**Time since diagnosis**							
<1 year	59(9.5)	18(16.7)	8(8.1)	9(8.4)	5(4.5)	14(12.4)	5(6.0)
1–2 years	176(28.3)	23(21.3)	14(14.1)	29(27.1)	45(40.2)	46(40.7)	19(22.9)
2–5 years	214(34.4)	37(34.3)	30(30.3)	34(31.8)	43(38.4)	38(33.6)	32(38.6)
>5years	173(27.8)	30(27.8)	47(47.5)	35(32.7)	19(17.0)	15(13.3)	27(32.5)
**Medicinal treatment for breast cancer**							

**Ever treatment**							
Tyrosine kinase inhibitor	42(6.7)	1(0.9)	2(2.1)	12(11.2)	8(7.1)	19(16.8)	0(0)
Signal transduction inhibitor	51(8.2)	5(4.6)	3(3.1)	12(11.2)	5(4.4)	26(23.0)	0(0)
Pan-HER inhibitor	38(6.1)	2(1.8)	1(1.1)	6(5.6)	4(3.5)	25(22.1)	0(0)
Monoclonal antibodies	493(79.3)	84(77.8)	73(73.7)	86(80.4)	87(77.7)	88(77.9)	75(90.4)
Antibody-drug conjugates	141(22.7)	18(16.7)	13(13.1)	25(23.4)	23(20.5)	45(39.8)	17(20.5)
Do not know	116(18.6)	18(16.7)	26(26.3)	16(15.0)	19(17.0)	20(17.7)	17(20.5)
**Current treatment**							
Yes	421(67.7)	70(64.8)	56(56.6)	74(69.2)	85(75.9)	88(77.9)	48(57.8)
No	198(31.8)	38(35.2)	43(43.4)	32(29.9)	27(24.1)	24(21.2)	34(41.0)
Do not know	3(0.5)	0(0)	0(0)	1(0.9)	0(0)	1(0.9)	1(1.2)

Data are given as N(%), BC; Breast Cancer.

a Includes employed or self-employed patients.

b Includes on sick leave, maternity or parental leave.

c Includes other or prefer not to state.

### 3.2 Worries and concerns about the health condition

**Fig. 2:**
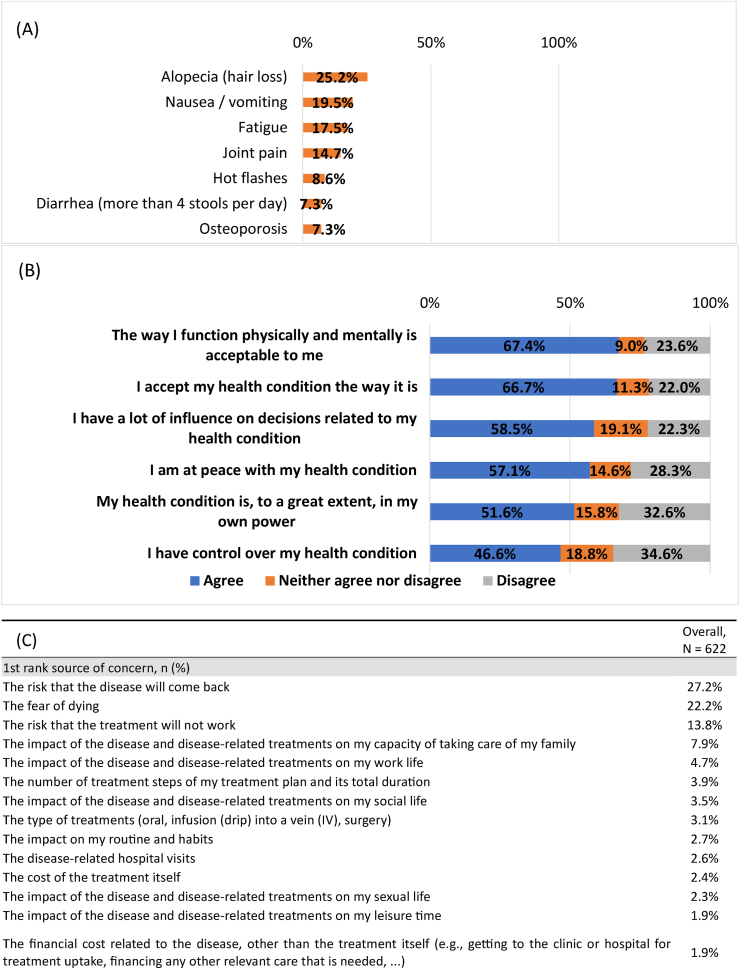
**Worries and concerns about the health condition (N=622)** (A) Difficult-to-manage side effects of breast cancer treatment. (B) Worries and concerns about the disease. (C) Worries and concerns about the treatment plan.

The side effects caused by anti-cancer treatments considered to be the least tolerable were alopecia (25.2 % ranked it first), followed by nausea/vomiting (19.5 %) and fatigue (17.5 %). The main toxicity-related concern was alopecia in patients with early BC (26.5 %) and fatigue in the metastatic setting (33.3 %) (Supplementary Table 2). Patients reported mixed feelings towards their condition with 22.0 % not agreeing that they have accepted their health condition the way it is, 23.6 % did not agree the way they were physically and mentally functioning is acceptable to them and 34.7 % believed they did not have control over their health condition. The main worries and concerns that were reported by patients were risk of recurrence (27.2 %), fear of dying (22.2 %), and risk of treatment failure (13.8 %) (Fig. 2).

Patients with early BC experienced numerically less physical and mental burden from the disease compared to those with metastatic BC (Supplementary Table 3).

#### 3.2.1 Patient interactions with healthcare professionals

Almost all patients (96.8 %) wanted to be involved in their treatment decision (49.2 % completely and 47.6 % partially). Patients expressed satisfaction with the duration of their consultations, with 53.2 % indicating high satisfaction levels. Overall, 30.4 % reported having fully discussed the risk of recurrence with their doctor, which was higher in metastatic BC compared to early BC (Supplementary Table 4), whereas 19.5 % did not discuss recurrence risk with their doctor. Most patients with early BC (72.1 %) favored short and simple explanations from the medical team when explaining the risk of cancer recurrence, while 4.2 % preferred not to receive this information (Table 2).

**Table 2:** Patient interactions with healthcare professionals and decision-making (N = 622).

	**N (%)**
**Involvement in the decisions regarding the treatment plan**	

Full Involvement^a^	306 (49.2)
Partial Involvement^b^	296 (47.6)
No Involvement^c^	20 (3.2)

**Satisfaction with the duration of medical team consultation**^d^	

High satisfaction	331 (53.2)
Moderate satisfaction	203 (32.6)
Low satisfaction	88 (14.1)

**Recurrence discussed with medical team**	

Yes, fully	189 (30.4)
Yes, partially	312 (50.2)
Not at all	121 (19.5)

**Preferred communication when explaining the risk of cancer recurrence** ∼	

Providing short explanations with simple words	380 (72.1)
Sharing numbers and statistics	209 (39.7)
Sharing other patients' experience	179 (34.0)
Showing you visuals (drawings/posters/videos)	124 (23.5)
Nothing, as I do not want any explanations from my medical team about the risk of cancer reappearing	22 (4.2)

∼ N = 527.

a I like to work closely with my healthcare team, and I prefer to take the decisions by myself.

b I like to be informed by my healthcare team, but I rely on their decisions.

c I do not need any information from my healthcare team, and I fully rely on their decisions.

d On a scale of 0–10, how much would you say the duration of your consultations with your medical team permits to cover your needs and questions concerning your breast cancer? Low satisfaction (score of 1–3); Moderate satisfaction (score 4–6); High satisfaction (score 7–10).

### 3.3 Patients’ perceived risk of recurrence

**Fig. 3:**
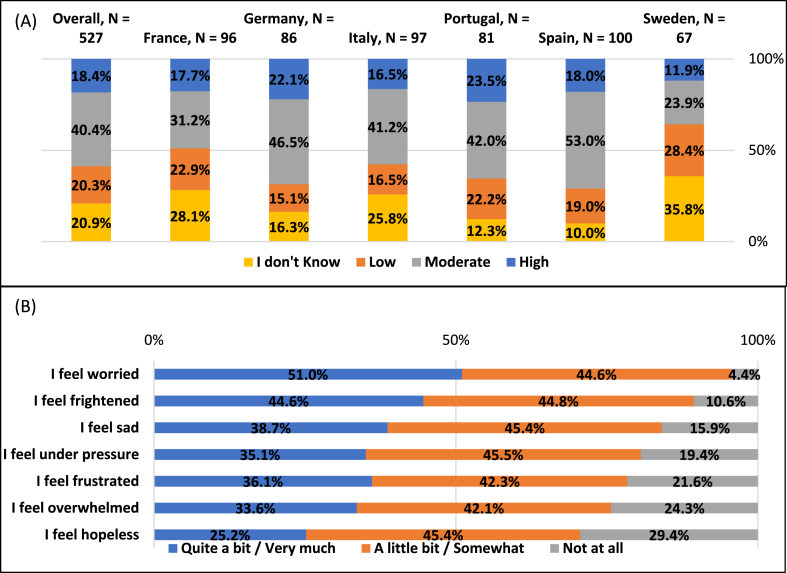
**Early breast cancer patients' perceived risk of breast cancer recurrence and associated feelings (awareness) (eBC; N=527)** (A) Perceived personal risk of breast cancer recurrence. (B) Patients' feelings when considering the risk of recurrence.

Patients with early BC perceived their personal risk of recurrence to be low (20.3 %), moderate (40.4 %), or high (18.4 %), while 20.9 % did not know their personal risk. Patients who were diagnosed with HER2+ early BC 1–2 years prior to the survey reported being more informed about their risk of recurrence (p < 0.001, Supplementary Table 5). When thinking about the risk of cancer recurrence, patients most commonly felt worried (51.0 % quite a bit/very much), frightened (44.6 %) or sad (38.7 %) (Fig. 3).

### 3.4 Patients’ lifestyle and medical choices

**Fig. 4:**
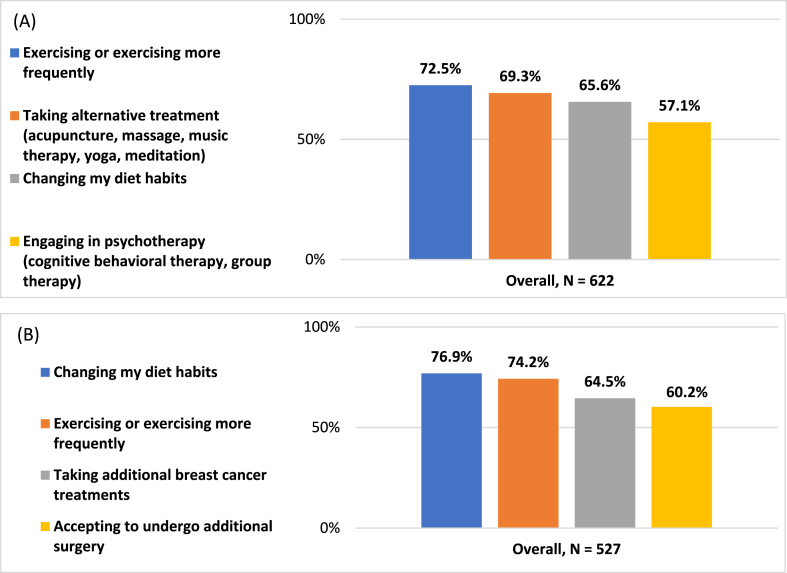
**Patients' lifestyle and medical choices.** (A) Preventive behaviors/changes to better manage the disease (N = 622). (B) Willingness of patients with early breast cancer to make lifestyle changes to reduce the risk of recurrence (eBC; N = 527).

To better manage the disease, 72.5 % of patients reported the willingness to increase their physical exercise frequency, 69.3 % were open to alternative treatments such as acupuncture or massage, and 65.6 % were willing to modify their dietary habits. Patients with early BC were statistically significantly more willing to change dietary habits and exercise compared to metastatic BC patients (p = 0.042 and p = 0.022, respectively). To decrease their risk of cancer recurrence, 76.9 % of patients were open to modifying their dietary habits, 74.2 % were willing to exercise more frequently, and 64.5 % were open to taking additional treatments for BC (Fig. 4).

Patients who wanted to be fully involved in decision making were statistically significantly more willing to accept additional treatment (p < 0.001, Supplementary Table 7).

#### 3.4.1 Additional treatment to reduce the risk of recurrence

**Fig. 5:**
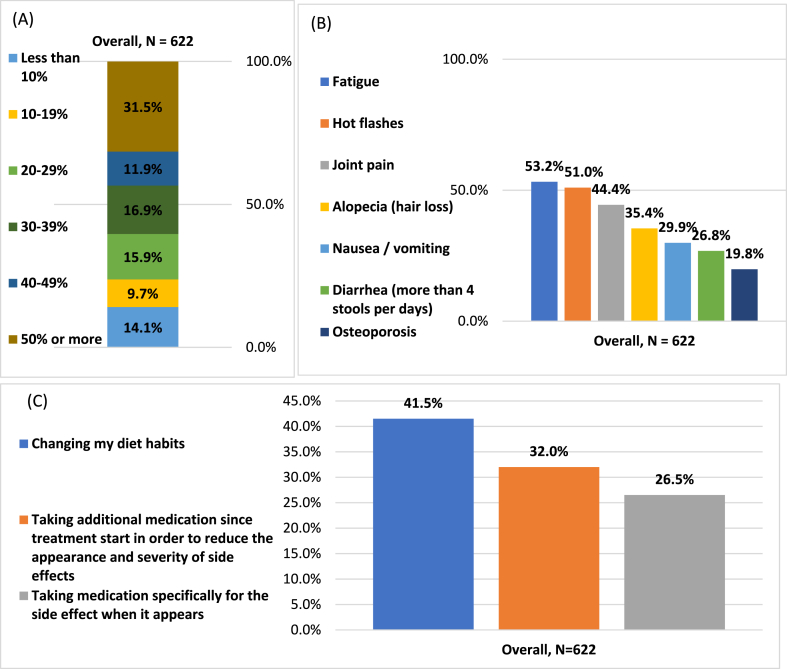
**Acceptance of additional interventions and treatments to reduce the risk of cancer recurrence (N=622).** (A) Acceptable efficacy for an additional treatment that reduces the recurrence risk. (B) Acceptable side effects for an additional treatment that reduces the recurrence risk. (C) Preventive behaviors to improve side effects of an additional treatment that reduces the recurrence risk.

A total of 68.5 % of patients were willing to accept an additional treatment that reduces the risk of cancer recurrence by less than 50 % (Fig. 5A). This was particularly pronounced among patients younger than 40 years (p < 0.001), diagnosed less than 2 years prior to the survey (p = 0.003), or who were willing to change their lifestyle (particularly their diet [p < 0.001]) (Supplementary Table 8). The motivation for an additional treatment that reduces the risk of recurrence reveals that the most tolerable side-effects for patients would have been fatigue (53.2 %), followed by hot flashes (51.0 %) and joint pain (44.4 %) (Fig. 5B). Changing diet habits was the most preventive behavior that the patients were willing to follow for improving side effects of an additional treatment that reduces the recurrence risk (41.5 %) (Fig. 5C).

Detailed results of each country are presented in Supplementary Tables 9–11. Results were consistent across countries.

## 4 Discussion

We report the results of a multinational direct-to-patient study examining the perceptions and concerns of women with HER2+ BC, particularly focusing on their risk of recurrence across six European countries. This survey covered three main aspects of women's experience of HER2+ BC: the psychological impact of the disease, knowledge and perceptions regarding their risk of recurrence, and their willingness to take actions to improve their condition.

Fear of recurrence or disease progression and death are important psychological burdens that affect the quality of life of patients with BC [14]. Although about half of patients felt psychologically at peace with their health condition and felt that they had control over it, the fear of disease recurrence, death, and treatment failure were identified as the most important concerns for approximately two-thirds of the patients. This is in line with previous studies showing similar levels of fear of disease recurrence among patients with early BC [14]. Another study conducted among 244 female patients with BC in China reported a negative correlation between fear of recurrence and quality of life [15]. In fact, fear of recurrence can diminish physical, mental, and social aspects of quality of life and can persist beyond completion of an active treatment. A psychological approach facilitating acceptance of the disease may improve patients' quality of life [16]. Patients’ trust in their physicians was associated with a reduction in their fears and anxiety; moreover, it increased satisfaction and adherence to treatment [17]. Therefore, it is imperative to psychologically support BC patients, manage their expectations and fears, and identify and alleviate their concerns starting from diagnosis throughout the cancer care trajectory.

Effective physician-patient communication is crucial in BC diagnosis and addressing the risk of recurrence provides comfort and high-quality care while positively influencing outcomes and treatment adherence [18]. In this survey, despite acceptable overall satisfaction with the length of medical consultations, one in five patients reported not discussing cancer recurrence with their healthcare providers. Interestingly, more than one in five patients were unaware of their individual risk of recurrence. This finding is consistent with previous studies showing that many patients with breast cancer either overestimate or underestimate their risk of recurrence [12,13,19], highlighting a critical gap in risk communication. Beyond the challenges associated with physicians' limited time, another factor influencing the inadequate communication of BC recurrence with patients is the challenges related to patients' numeracy [20], with patients having difficulties in understanding and interpreting risk numbers. This study also explored the preferred way to communicate about recurrence: notably, most of the patients favored short and simple explanations. Our study provides further evidence that patients with BC generally report a lack of communication and perceive that they have insufficient information on their risk of recurrence [12,21]. Patients highly engaged in treatment planning often initiate discussions about risk of recurrence and supplement their knowledge with their own research [10]. Efforts need to be made to enhance physicians’ ability to engage in individualized communication around risk [21]. In a qualitative study, patients with BC highlighted the need for effective communication with their HCPs based on trust and ethical standards [22]. A clear and balanced dialogue is needed to manage patient expectations.

Almost all patients expressed their willingness to be involved in decision making, desiring to be either partially or fully involved in discussions around their treatment plan. This shared decision-making approach not only enhances patients’ satisfaction with decisions, reduces stress, and improves adherence to anticancer treatment [23], but also empowers patients to make more informed choices to decrease the risk of cancer recurrence, facilitating better communication and understanding of this risk [24]. It is crucial for physicians to be aware that most of the patients prefer to be actively engaged in the decision-making process. Pharmacogenomic and genomic testing in oncology can be valuable tools to guide patients and physicians in decision making. These tools are particularly useful in predicting treatment response, toxicity risk, and long-term outcomes, thereby optimizing the risk-benefit ratio of therapeutic interventions [25]. Genomic screening and testing for specific markers may allow early identification of high-risk patients, allowing clinicians to tailor treatment regimens by adjusting dosages, incorporating protective agents, or considering alternative therapies. Although challenges associated with pharmacogenetic and genomic testing include the cost of testing, interpretation of genetic data, and integration of pharmacogenomics into clinical decisions, the potential of pharmacogenomics to optimize decision making remains an evolving field with growing importance in HER2+ BC management.

Patients identified alopecia as the most difficult-to-manage side effect of BC treatment followed by nausea/vomiting and fatigue. Previous studies revealed that 17 % of women with gynaecological cancers reported alopecia as the most traumatic adverse event during treatment, 30 % reported that they were severely limited by it, and up to 14 % would have considered rejecting curative therapies associated with alopecia [24,[26], [27], [28], [29]]. Another study among women with BC who had endocrine therapy-induced alopecia expressed it as the most disturbing anticipated adverse event leading to high psychological distress [26]. Gastrointestinal toxicities and general fatigue were also shown to have a negative effect [28]. No information on hormone receptor status and use of endocrine therapy was captured in our survey; thus, their impact on our results cannot be assessed.

In the present survey, most patients with non-metastatic cancer reported their willingness to change dietary habits, to exercise, to accept additional BC treatments and expressed being ready to undergo additional surgery to reduce the risk of cancer recurrence. Previous studies demonstrated that cancer survivors engaged proactively in healthy lifestyles (e.g., abstaining from alcohol and tobacco and engaging in routine exercise) when they received support or were encouraged by their social support networks to change their habits [30,31]. Fear of death or treatment failure, anxiety, emotional distress, and frustration may motivate patients to modify their lifestyles. The findings of the present survey are consistent with previous reports showing that dealing with a life-threatening disease encourages patients to cease unhealthy habits and adopt healthier lifestyles, specifically in terms of dietary patterns and regular physical exercise [32,33].

New anticancer therapies have increased treatment options and survival rates but at the cost of additional side effects. In our survey, patients were willing to tolerate side effects of a potential new treatment that reduces the risk of recurrence; fatigue and hot flashes were the most acceptable side effects. In addition, a high percentage of patients (64.5 %) were willing to take an additional BC treatment even if the treatment efficacy is less than 50 %. Our results are in line with previous studies on patient preferences: in a recent systematic literature review of 34 studies, respondents considered treatment efficacy (in terms of overall or progression-free survival) as the most important characteristic (compared to side effects, cost of treatment, and route of administration), and women in particular considered small benefits (i.e. an additional year of life or 3–5 % increase in survival) to be sufficient to make (adjuvant) chemotherapy worthwhile [34]. Another study found that even in the absence of evidence of additional clinical benefit, more than one-third of patients already receiving chemotherapy would accept an additional treatment [35].

Although we found that women are willing to engage in several beneficial lifestyle behaviours to reduce the risk of recurrence, willingness does not necessarily translate into action. It is important to not only be willing to engage in beneficial lifestyle behaviours but also to take steps to ensure that these changes are implemented. This can include setting achievable goals, seeking support from caregivers and patient organizations, and making gradual changes to patients’ lifestyles.

### 4.1 Strengths and limitations

This study was conducted with a relatively large sample size, and recruitment was achieved through different sources using regional soft quotas, optimizing the generalizability of results to the entire population of each country. However, relying on convenience sampling methods, especially online, could introduce selection bias, potentially excluding individuals with limited or no internet access or literacy skills. Patients' willingness and ability to participate might affect representativeness, despite the efforts to minimize biases through soft quotas. Self-reported data can also introduce potential biases, including social desirability, inaccurate memory which could result in recall errors, intentional or unintentional false reporting, and difficulty in fully understanding the questionnaire. HER2 status was not reported by physicians but was self-reported by patients, which may have introduced inaccuracies if patients misunderstood their disease characteristics. This multinational study included patients from countries with different healthcare systems and cultural norms; these factors, in addition to socioeconomic status, may have influenced patients’ perceptions. Exploring the impact of this variability was outside the scope of this study but it may have affected the robustness of the conclusions. Furthermore, the heterogeneity of the patient population in terms of disease stage and time since diagnosis may impact upon the generalizability of the results. Several statistically significant differences were identified between sub-groups (e.g., duration since initial diagnosis of HER2+ BC). Whilst these findings may help to focalize future efforts to enhance patient awareness and provide optimal support, it is important to differentiate between statistical significance and clinical relevance. Effect sizes which could provide an indication of the magnitude of the relationship between variables were not calculated. The practical relevance and implications of these results need further investigation.

### 4.2 Conclusions

The present study emphasizes the crucial need for healthcare providers to proactively involve patients with early and metastatic HER2+ BC in their treatment decisions. A more open communication from the beginning of a patient's treatment journey is warranted to establish confidence for the entire treatment, either in early BC with the most likely chance of curing the disease as likewise towards the promising new therapies in mBC to avoid treatment attrition. Moreover, women expressed readiness to adopt healthier lifestyles and accept additional treatments for improving care and reducing the risk of recurrence. Enhancing patient communication should be considered with the aims to ultimately facilitate increased participation in shared decision-making and adherence to proposed interventions throughout their journey with the disease. Patient perception on risk of recurrence and decision-making in the management of HER2-positive early BC should be actively and routinely monitored locally in the clinical practice so that corrective measures can be put in place. This could be achieved by optimizing patient-physician communication through broader implementation of communication skills training for physicians, the increased use of short explanations using easy-to-understand vocabulary to provide personal risk information to patients in a less complex manner, and increased coordination between multidisciplinary teams.

## CRediT authorship contribution statement

**Matteo Lambertini:** Writing – review & editing, Validation, Supervision. **Christian Jackisch:** Writing – review & editing, Validation. **Olivier Trédan:** Writing – review & editing, Validation, Conceptualization. **Maria Vidal:** Writing – review & editing, Validation. **Mário Fontes-Sousa:** Writing – review & editing, Validation. **Antonios Valachis:** Writing – review & editing, Validation. **Rosanna D'Antona:** Writing – review & editing, Validation. **Marcelo Ruz:** Writing – review & editing, Validation. **Eugenia Krone:** Writing – review & editing, Validation. **Miriam Brice:** Writing – review & editing, Validation. **Erwan Berjonneau:** Writing – review & editing, Project administration, Validation, Conceptualization. **Soraia Matos:** Supervision, Methodology, Conceptualization, Validation. **Olivia Dialla:** Validation, Methodology. **Laure Guéroult-Accolas:** Writing – review & editing, Validation.

## Funding

Pierre Fabre funded the study: its design, data collection, analysis and interpretation. Pierre Fabre also funded the development (writing and submission) of this manuscript.

## Declaration of competing interest

• ML reports advisory role for Roche, Lilly, Menarini, Novartis, Astrazeneca, Pfizer, Seagen, Gilead, MSD, Exact Sciences, Pierre Fabre; speaker honoraria from Roche, Lilly, Menarini, Novartis, Pfizer, Sandoz, Libbs, Daiichi Sankyo, Takeda, Gilead; travel Grants from Gilead, Daiichi Sankyo, Roche; research funding (to the Institution) from Gilead all outside the submitted work.
• AV reports institutional unrestricted grants from Roche and MSD. Also reports advisory role for Pierre Fabre.
• OT reports having received honoraria from Pierre Fabre, Roche, Pfizer, Novartis-Sandoz, Lilly, MSD, Astra-Zeneca, Seagen, Daiichi-Sankyo, Gilead, Eisai, Menarini-Stemline and Veracyte.
• CJ reports advisory role for Roche, Lilly, Novartis, Astrazeneca, Pfizer, Seagen, Gilead, MSD, Exact Sciences, Pierre Fabre; speaker honoraria from Roche, Lilly, Novartis, Pfizer, Daiichi Sankyo, Gilead; travel Grants from Gilead, Daiichi Sankyo, Roche; all outside the submitted work.
• MFS reports advisory role or speaker honoraria for Astellas, AstraZeneca, Bayer, Bristol Myers Squibb, Daiichi-Sankyo, Gilead, Ipsen, Lilly, Merck, Merck Sharp & Dohme, Novartis, Pfizer. Also reports advisory role for Pierre Fabre.
• MV, RA, MR, EK, MB, LGA received compensation from Pierre Fabre for advisory role.
• SM, OD are employees of the sponsor, Pierre Fabre.
• EB works at Oracle Life Science.
