# Supplementary material for: Patient perception on risk of recurrence and decision-making in the management of HER2-positive early breast cancer: Insights from the ASKHER2 European survey
Source: Breast. 2025 Mar 21;81:104456. doi: 10.1016/j.breast.2025.104456 (PMC11992529; doi:10.1016/j.breast.2025.104456)
Supplement: Multimedia component 1 [file mmc1.docx]

**Title:** **Patient Perception on Risk of Recurrence and Decision-Making in the management of HER2-positive Early Breast Cancer: Insights from the ASKHER2 European Survey**

**Supplementary material**

|  | **France** | **Germany** | **Spain** | **Italy** | **Portugal** | **Sweden** |
| --- | --- | --- | --- | --- | --- | --- |
| **Online patient panels*** | 49 | 51 | 111 | 51 | 50 | - |
| **PAG*** | 59 | 48 | 2 | 56 | 62 | 83 |
| **Total** | 108 | 99 | 113 | 107 | 112 | 83 |
| *Soft quotas were applied to achieve the total target sample size per country  PAG; Patient Advocacy Groups | | | | | | |

**Supplementary Table 1. Achieved sample size of the 622 patients across countries**

| **Symptoms** | **eBC**  **(N= 514)** | **mBC**  **(N=72)** | **p-value** |
| --- | --- | --- | --- |
| **Alopecia** | 136(26.5) | 14(19.4) |  |
| **Diarrhea*** | 38(7.4) | 5(6.9) |  |
| **Fatigue** | 79(15.4) | 24(33.3) | 0.02836 |
| **Hot flashes** | 49(9.5) | 3(4.2) |  |
| **Joint pain** | 77(15.0) | 7(9.7) |  |
| **Nausea/vomiting** | 98(19.1) | 15(20.8) |  |
| **Osteoporosis** | 37(7.2) | 4(5.6) |  |
| Data are given as N(%)  *More than 4 stools per day  eBC=Early breast cancer, mBC=Metastatic breast cancer | | |  |

**Supplementary Table 2. Perception of difficult-to-manage side effect(s) of breast cancer treatments**

|  | **eBC**  **(N= 527)** | **mBC**  **(N=74)** | **p-value** |
| --- | --- | --- | --- |
| **I am at peace with my health condition** | | | |
| Agree | 312 (59.2) | 33 (44.6) |  |
| Neither agree nor disagree | 73 (13.9) | 15 (20.3) | 0.12 |
| Disagree | 142 (26.9) | 26 (35.1) |  |
| **I have the feeling that I have control over my health condition** | | | |
| Agree | 252 (47.8) | 30 (40.5) |  |
| Neither agree nor disagree | 99 (18.8) | 14 (18.9) | 0.6 |
| Disagree | 176 (33.4) | 30 (40.5) |  |
| **I have the feeling that I have a lot of influence on decisions related to my health condition** | | | |
| Agree | 311 (59.0) | 42 (56.8) |  |
| Neither agree nor disagree | 92 (17.5) | 20 (27.0) | 0.11 |
| Disagree | 124 (23.5) | 12 (16.2) |  |
| **The way in which I am functioning physically and mentally is acceptable to me** | | | |
| Agree | 363 (68.9) | 40 (54.1) |  |
| Neither agree nor disagree | 46 (8.7) | 8 (10.8) | 0.086 |
| Disagree | 118 (22.4) | 26 (35.1) |  |
| **I accept my health condition the way it is** | | | |
| Agree | 364 (69.1) | 40 (54.1) |  |
| Neither agree nor disagree | 51 (9.7) | 15 (20.3) | 0.018 |
| Disagree | 112 (21.3) | 19 (25.7) |  |
| **My health condition is, to a great extent, in my own power** | | | |
| Agree | 281 (53.3) | 27 (36.5) |  |
| Neither agree nor disagree | 85 (16.1) | 10 (13.5) | 0.017 |
| Disagree | 161 (30.6) | 37 (50.0) |  |
| Data are given as N(%)  eBC=Early breast cancer, mBC=Metastatic breast cancer | | | |

**Table supplementary 3. Worries and concerns about the disease**

| **Supplementary Table 4. Communication with clinician about risk of recurrence** | | | |
| --- | --- | --- | --- |
|  | **eBC**  **(N= 527)** | **mBC**  **(N=74)** | **P value** |
| **Fully** | 149 (28.3) | 37 (50.0) | <0.001 |
| **Partially** | 273 (51.8) | 30 (40.5) |  |
| **No** | 105 (19.9) | 7 (9.5) |  |
| Data are given as N(%)  eBC=Early breast cancer, mBC=Metastatic breast cancer | | | |

| **Supplementary Table 5. Early breast cancer patients’ perceived risk of breast cancer recurrence** | | | | | |
| --- | --- | --- | --- | --- | --- |
|  | **Duration since initial diagnosis of HER2+ breast cancer** | | | | |
|  | **Less than 1 year**  **(N=56)** | **1-2 years**  **(N=146)** | **2-5 years**  **(N=185)** | **More than 5 years**  **(N=140)** | **P value** |
| **Low** | 16 (28.6) | 21 (14.4) | 36 (19.5) | 34 (24.3) | <0.001 |
| **Moderate** | 10 (17.9) | 79 (54.1) | 75 (40.5) | 49 (35.0) |  |
| **High** | 9 (16.1) | 26 (17.8) | 35 (18.9) | 27 (19.3) |  |
| **Don’t know** | 21 (37.5) | 20 (13.7) | 39 (21.1) | 30 (21.4) |  |
| Data are given as N(%) | | | | | |

| **Supplementary Table 6. Willingness of early breast cancer patients to make lifestyle changes to reduce the risk of recurrence** | | | | | |
| --- | --- | --- | --- | --- | --- |
|  | **Perceived personal risk of breast cancer recurrence** | | | | |
|  | **Low**  **(N=107)** | **Moderate**  **(N=213)** | **High**  **(N=97)** | **Don’t know**  **(N=110)** | **p-value** |
| **Changing my diet habits** | 86 (80.4) | 159 (74.6) | 77 (79.4) | 83 (75.5) | 0.6 |
| **Exercising or exercising more frequently** | 86 (80.4) | 146 (68.5) | 74 (76.3) | 85 (77.3) | 0.092 |
| **Accepting to undergo additional surgery** | 53 (49.5) | 128 (60.1) | 66 (68.0) | 70 (63.6) | 0.044 |
| **Taking additional breast cancer treatments** | 64 (59.8) | 132 (62.0) | 66 (68.0) | 78 (70.9) | 0.2 |
| **Other** | 5 (4.7) | 18 (8.45) | 7 (7.2) | 13 (11.8) | 0.3 |
| **None of the above** | 6 (5.6) | 4 (1.9) | 2 (2.1) | 1 (0.9) | 0.2 |
| Data are given as N(%) | | | | |  |

| **Supplementary Table 7. Willingness of early breast cancer patients to make lifestyle changes to reduce the risk of recurrence** | | | | |
| --- | --- | --- | --- | --- |
|  | **Willingness to be involved in your treatment plan decision-making** | | | |
|  | **No information preferred, fully reliant on healthcare team for decisions**  **(N=19)** | **Information preferred, reliant on healthcare team for decisions**  **(N=256)** | **Full involvement on information and decisions preferred**  **(N=252)** | **P value** |
| **Changing my diet habits** | 16 (84.2) | 196 (76.6) | 193 (76.6) | 0.8 |
| **Exercising or exercising more frequently** | 16 (84.2) | 191 (74.6) | 184 (73.0) | 0.6 |
| **Accepting to undergo additional surgery** | 16 (84.2) | 147 (57.4) | 154 (61.1) | 0.065 |
| **Taking additional breast cancer treatments** | 5 (26.3) | 160 (62.5) | 175 (69.4) | <0.001 |
| **Other** | 0 (0) | 20 (7.8) | 23 (9.1) | 0.5 |
| **None of the above** | 1 (5.3) | 4 (1.6) | 8 (3.2) | 0.2 |
| Data are given as N(%) | | | |  |

| **Supplementary Table 8. Willingness to accept additional treatment with an efficacy less or more than 50%** | | | |
| --- | --- | --- | --- |
|  | **Less than 50%**  **(N=426)** | **50% and more**  **(N=196)** | **P value** |
| **Age group** |  |  | <0.001 |
| 18-39 | 115 (27.0) | 18 (9.2) |  |
| 40-65 | 283 (66.4) | 152 (77.6) |  |
| Above 65 years old | 28 (6.6) | 26 (13.3) |  |
| **Duration since initial diagnosis of HER2+ breast cancer** |  |  | 0.003 |
| Less than 1 year | 41 (9.6) | 18 (9.2) |  |
| 1-2 years | 136 (31.9) | 40 (20.4) |  |
| 2-5 years | 147 (34.5) | 67 (34.2) |  |
| More than 5 years | 102 (23.9) | 71 (36.2) |  |
| eBC | **Less than 50%**  **(N=362)** | **50% and more**  **(N=165)** | **P value** |
| **Lifestyle changes*** |  |  |  |
| Changing my diet habits | 295 (81.5) | 110 (66.7%) | <0.001 |
| Exercising or exercising more frequently | 279 (77.1) | 112 (67.9%) | 0.025 |
| Accepting to undergo additional surgery | 227 (62.7) | 90 (54.5%) | 0.076 |
| Taking additional breast cancer treatments | 239 (66.0) | 101 (61.2%) | 0.3 |
| Data are given as N(%)  *Only asked to eBC patients | | | |

| **Supplementary Table 9. Patient interactions with healthcare professionals and decision-making per country** | | | | | | | |
| --- | --- | --- | --- | --- | --- | --- | --- |
| **Involvement in the decisions regarding the treatment plan** | | | | | | | |
|  | **France**  **(N=108)** | **Germany**  **(N=99)** | **Italy**  **(N=107)** | | **Portugal**  **(N=112)** | **Spain**  **(N=113)** | **Sweden**  **(N=83)** |
| **Full^1^** | 45(41.7) | 60(60.6) | 54(50.5) | | 70(62.5) | 47(41.6) | 30(36.1) |
| **Partial^2^** | 60(55.6) | 38(38.4) | 51(47.7) | | 41(36.6) | 53(46.9) | 53(63.9) |
| **No^3^** | 3(2.8) | 1(1.0) | 2(1.8) | | 1(0.9) | 13(11.5) | 0(0) |
| **Satisfaction with the duration of medical team consultation*** | | | | | | | |
| **High** | 53(49.1) | 51(51.5) | 75(70.1) | | 65(58.0) | 57(50.4) | 30(36.1) |
| **Moderate** | 34(31.5) | 32(32.3) | 25(23.4) | | 34(30.4) | 40(35.4) | 38(45.8) |
| **Low** | 21(19.4) | 16(16.2) | 7(6.5) | | 13(11.6) | 16(14.2) | 15(18.1) |
| **Recurrence discussed with medical team** | | | | | | | |
| **Yes, fully** | 23(21.3) | 31(31.3) | 30(28.0) | | 45(40.2) | 52(46.0) | 8(9.6) |
| **Yes, partially** | 51(47.2) | 51(51.5) | 59(55.1) | | 50(44.6) | 55(48.7) | 46(55.4) |
| **Not at all** | 34(31.5) | 17(17.2) | 18(16.8) | | 17(15.2) | 6(5.3) | 29(34.9) |
| **Preferred communication when explaining the risk of cancer recurrence**** | | | | | | | |
| **eBC** | **France**  **(N=96)** | **Germany**  **(N=86)** | **Italy**  **(N=97)** | **Portugal**  **(N=81)** | | **Spain**  **(N=100)** | **Sweden**  **(N=67)** |
| **Providing short explanations with simple words** | 69(71.9) | 70(81.4) | 69(71.1) | | 53(65.4) | 84(84.0) | 35(52.2) |
| **Sharing numbers and statistics** | 34(35.4) | 35(40.7) | 41(42.3) | | 28(34.6) | 32(32.0) | 39(58.2) |
| **Sharing other patients’ experience** | 38(39.6) | 25(29.1) | 33(34.0) | | 29(35.8) | 43(43.0) | 11(16.4) |
| **Showing you visuals (drawings / posters / videos)** | 9(9.4) | 19(22.1) | 26(26.8) | | 16(19.8) | 33(33.0) | 21(31.3) |
| **Nothing, as I do not want any explanations from my medical team about the risk of cancer reappearing** | 9(9.4) | 4(4.6) | 3(3.1) | | 4(4.9) | 0(0) | 2(3.0) |
| Data are given as N(%)  ^1^I like to work closely with my healthcare team, and I prefer to take the decisions by myself  ^2^I like to be informed by my healthcare team, but I rely on their decisions  ^3^I do not need any information from my healthcare team, and I fully rely on their decisions  *****On a scale of 0 to 10, how much would you say the duration of your consultations with your medical team permits to cover your needs and questions concerning your breast cancer? Low satisfaction (score of 1-3); Moderate satisfaction (score 4-6); High satisfaction (score 7-10)  **Only asked to eBC patients | | | | | | | |

| **Supplementary Table 10.** **Perception of difficult-to-manage side effects of breast cancer treatments per country** | | | | | | |
| --- | --- | --- | --- | --- | --- | --- |
|  | **France**  **(N = 108)** | **Germany**  **(N = 99)** | **Italy**  **(N = 107)** | **Portugal**  **(N=112)** | **Spain**  **(N=113)** | **Sweden**  **(N=83)** |
| **1st rank side effect, n (%)** | | | | | | |
| **Alopecia** | 33(31.4) | 16(16.5) | 30(28.6) | 26(23.9) | 29(25.9) | 19(24.4) |
| **Diarrhea*** | 1(0.9) | 9(9.3) | 10(9.5) | 7(6.4) | 11(9.8) | 6(7.7) |
| **Fatigue** | 31(29.5) | 20(20.6) | 7(6.7) | 22(20.2) | 11(9.8) | 15(19.2) |
| **Hot flashes** | 11(10.5) | 10(10.3) | 9(8.6) | 9(8.3) | 10(8.9) | 3(3.8) |
| **Joint pain** | 7(6.7) | 17(17.5) | 17(16.2) | 18(16.5) | 22(19.6) | 8(10.3) |
| **Nausea / vomiting** | 19(18.1) | 19(19.6) | 22(21.0) | 20(18.3) | 16(14.3) | 22(28.2) |
| **Osteoporosis** | 3(2.9) | 6(6.2) | 10(9.5) | 7(6.4) | 13(11.6) | 5(6.4) |
| Data are given as N(%)  *More than 4 stools per day | | | | | | |

| **Supplementary Table 11. Worries and concerns about the disease per country** | | | | | | |
| --- | --- | --- | --- | --- | --- | --- |
|  | **France**  **(N = 108)** | **Germany**  **(N = 99)** | **Italy**  **(N = 107)** | **Portugal**  **(N=112)** | **Spain**  **(N=113)** | **Sweden**  **(N=83)** |
| **I am at peace with my health condition, n (%)** | | | | | | |
| **Agree** | 48(44.4) | 56(56.6) | 67(62.6) | 66(58.9) | 60(53.1) | 58(69.9) |
| **Neither agree nor disagree** | 22(20.4) | 12(12.1) | 17(15.9) | 14(12.5) | 18(15.9) | 8(9.64) |
| **Disagree** | 38(35.2) | 31(31.3) | 23(21.5) | 32(28.6) | 35(31.0) | 17(20.5) |
| **I have the feeling that I have control over my health condition, n (%)** | | | | | | |
| **Agree** | 42(38.9) | 48(48.5) | 50(46.7) | 48(42.9) | 55(48.7) | 47(56.6) |
| **Neither agree nor disagree** | 25(23.1) | 20(20.2) | 28(26.2) | 14(12.5) | 17(15.0) | 13(15.7) |
| **Disagree** | 41(38.0) | 31(31.3) | 29(27.1) | 50(44.6) | 41(36.3) | 23(27.7) |
| **I have the feeling that I have a lot of influence on decisions related to my health condition, n (%)** | | | | | | |
| **Agree** | 58(53.7) | 59(59.6) | 61(57.0) | 69(61.6) | 69(61.1) | 48(57.8) |
| **Neither agree nor disagree** | 21(19.4) | 20(20.2) | 22(20.6) | 17(15.2) | 15(13.3) | 24(28.9) |
| **Disagree** | 29(26.9) | 20(20.2) | 24(22.4) | 26(23.2) | 29(25.7) | 11(13.3) |
| **The way in which I am functioning physically and mentally is acceptable to me, n (%)** | | | | | | |
| **Agree** | 70(64.8) | 64(64.6) | 84(78.5) | 64(57.1) | 77(68.1) | 60(72.3) |
| **Neither agree nor disagree** | 12(11.1) | 12(12.1) | 6(5.61) | 14(12.5) | 9(8.0) | 3(3.6) |
| **Disagree** | 26(24.1) | 23(23.2) | 17(15.9) | 34(30.4) | 27(23.9) | 20(24.1) |
| **I accept my health condition the way it is, n (%)** | | | | | | |
| **Agree** | 70(64.8) | 64(64.6) | 77(72.0) | 65(58.0) | 80(70.8) | 59(71.1) |
| **Neither agree nor disagree** | 12(11.1) | 7 (7.1) | 13(12.1) | 19 (17.0) | 7 (6.19) | 12(14.5) |
| **Disagree** | 26(24.1) | 28(28.3) | 17(15.9) | 28(25.0) | 26(23.0) | 12(14.5) |
| **My health condition is, to a great extent, in my own power, n (%)** | | | | | | |
| **Agree** | 47(43.5) | 62(62.6) | 49(45.8) | 55(49.1) | 63(55.8) | 45(54.2) |
| **Neither agree nor disagree** | 20(18.5) | 13(13.1) | 22(20.6) | 17(15.2) | 14(12.4) | 12(14.5) |
| **Disagree** | 41(38.0) | 24(24.2) | 36(33.6) | 40(35.7) | 36(31.9) | 26(31.3) |
| Data are given as N(%) | | | | | | |
